# Plasma biomarker strategies to reduce diagnostic uncertainty in p-tau217–based assessment of CSF amyloid positivity in secondary-care patients with MCI

**DOI:** 10.3389/fnagi.2026.1918304

**Published:** 2026-09-28

**Authors:** María Pascual-Lucas, Leticia Sarasa, Noelia Fandos, Jorge Loscos, Judith Romero, Adrián Sánchez, Jose Terencio, Isabel Ortega-Madueño, María Cruz Cárdenas, Fernando Cava-Valenciano, María José Gil-Moreno, Jordi A. Matias-Guiu, José Antonio Allué

**Affiliations:** 1 Araclon Biotech-Grifols, Zaragoza, Spain; 2 Scientific Innovation Office, Grifols S.A., Sant Cugat del Vallès, Spain; 3 Department of Clinical Analysis, Institute of Laboratory Medicine, Hospital Clínico San Carlos, San Carlos Health Research Institute (IdISSC), Universidad Complutense de Madrid, Madrid, Spain; 4 Department of Neurology, Hospital Clínico San Carlos, San Carlos Health Research Institute (IdISSC), Universidad Complutense de Madrid, Madrid, Spain

**Keywords:** ABtest-MS, Alzheimer’s disease, Aβ42/Aβ40, grey zone, Lumipulse, multimarker models, p-tau217, plasma biomarkers

## Abstract

**Introduction:**

Plasma p-tau217 accurately reflects amyloid pathology, but dual-cutoff strategies leave a non-negligible grey zone requiring confirmatory testing. We evaluated whether integrating plasma Aβ measurements could reduce diagnostic uncertainty in patients with mild cognitive impairment (MCI).

**Methods:**

Plasma p-tau217 was measured using the Lumipulse platform, and plasma Aβ40 and Aβ42 were quantified by liquid chromatography-mass spectrometry (ABtest-MS) in 186 secondary-care MCI patients. CSF Aβ42/Aβ40 defined amyloid status. Logistic regression models were compared, and dual-cutoff strategies targeting 95% predictive values were applied.

**Results:**

p-tau217 was the strongest individual biomarker. Models incorporating plasma Aβ measures improved AUC, model fit, and reduced the grey zone from 27.4% with p-tau217 alone to 12.3%. A sequential two-step strategy restricted additional Aβ testing to p-tau217-indeterminate cases and reduced the final grey zone to 13.4%.

**Discussion:**

Plasma Aβ measurements complement p-tau217 by reducing the indeterminate results, with the potential to improve diagnostic efficiency and streamline diagnostic workflows.

## 1 Introduction

Blood-based biomarkers have rapidly transitioned from research tools to diagnostic tests suitable for real-world clinical use in the identification of cerebral amyloid-beta (Aβ) pathology in Alzheimer’s disease (AD). Among currently available plasma markers, phosphorylated tau at threonine 217 (p-tau217) has consistently demonstrated the highest diagnostic accuracy across cognitively impaired individuals, with reported performance comparable to cerebrospinal fluid (CSF) analysis (Janelidze et al., 2023; Ashton et al., 2024; Barthélemy et al., 2024; Mattsson-Carlgren et al., 2024; Meyer et al., 2024; Palmqvist et al., 2024; Schindler et al., 2024b; Palmqvist et al., 2025a; Warmenhoven et al., 2025).

Nevertheless, in clinical practice, plasma p-tau217 is commonly implemented using dual-cutoff strategies (Coste and Pouchot, 2003) aimed at maximizing diagnostic reliability by imposing high sensitivity and specificity thresholds, typically ≥90%. This approach inevitably creates an intermediate “grey zone,” frequently exceeding 20% of tested individuals (Figdore et al., 2024; Wilson et al., 2025a). Patients falling within this indeterminate range cannot be confidently classified as positive or negative, and therefore, require confirmatory CSF analysis or amyloid PET imaging (Brum et al., 2023). Although plasma testing represents a major advance and substantially streamlines diagnostic pathways for many patients, the grey zone remains a relevant limitation in routine clinical practice. It continues to generate uncertainty for clinicians and leads to additional invasive or costly procedures for a considerable subset of individuals.

In this context, strategies that integrate additional biomarkers represent a promising approach to enhance classification performance and reduce grey zone results. Plasma Aβ, particularly, provides complementary biological information that directly reflects cerebral amyloid burden (Mattsson-Carlgren et al., 2021).

Consistent with this rationale, the U.S. Food and Drug Administration has recently cleared a plasma p-tau217/Aβ42 ratio measured on the Lumipulse platform (Fujirebio, Tokyo, Japan) to aid in the detection of amyloid pathology (Tanne, 2025). While this represents an important step toward routine clinical implementation, ratio-based diagnostic approaches inherently rely on the analytical precision and robustness of both individual biomarkers.

Plasma Aβ42 has long been recognized as a particularly challenging analyte (Pérez-Grijalba et al., 2016) due to its low circulating concentrations, susceptibility to preanalytical variability, and the known limitations of immunoassay-based measurements. Immunoassays have historically shown higher inter-assay variability and matrix effects compared to mass spectrometry (MS)–based methods (Verberk et al., 2018; Klafki et al., 2022; Janelidze et al., 2023).

In this study, we evaluated multiple diagnostic scenarios in individuals with mild cognitive impairment (MCI) by integrating plasma p-tau217, measured using the Lumipulse platform, with Aβ peptides quantified by ABtest-MS, an antibody-free targeted liquid chromatography-MS (LC–MS) method optimized for high analytical precision. In addition to assessing each biomarker individually, we explored composite approaches combining both markers. Our objective was not only to maximize overall diagnostic accuracy, but also to achieve a clinically meaningful reduction in indeterminate results and the need for CSF analyses or amyloid PET scans, while maintaining high sensitivity and specificity. Based on these analyses, we propose practical diagnostic workflows to streamline patient assessment and improve overall efficiency in real-world clinical settings, where cost, accessibility, and patient burden are critical considerations.

## 2 Materials and methods

### 2.1 Participants in the study

Plasma samples from 186 participants prospectively recruited in the memory unit of Hospital Clínico Universitario San Carlos (HCSC, Madrid, Spain) between January 2022 and May 2025 were included in this study. Patients were consecutively enrolled based on a clinical diagnosis of MCI in whom lumbar puncture for CSF biomarker study was considered clinically indicated as part of the diagnostic work-up, and who agreed to undergo the procedure. All patients meeting the eligibility criteria during the recruitment period and with CSF and blood samples were included. Inclusion criteria were: (a) presence of cognitive symptoms; (b) preserved functional status in daily-living activities, defined as a Functional Activities Questionnaire score <11 (i.e., not meeting criteria for dementia); and (c) objective cognitive impairment (Z-score <−1 in at least one cognitive test, adjusted for age and education), consistent with the National Institute on Aging and Alzheimer’s Association workgroups (NIA-AA) criteria for MCI (Albert et al., 2011). Detailed information on the neuropsychological assessment for the definition of cognitive impairment is provided in Supplementary methods. The only exclusion criterion was contraindication to lumbar puncture.

Of the 200 participants initially included, 14 were subsequently excluded because of missing biomarker or APOE data or analytical quality-control failure, resulting in a final cohort of 186 participants. Plasma and CSF samples were collected on the same day, with an interval of no more than 1 h between collections. Laboratory personnel performing the plasma biomarker assays were blinded to CSF biomarker results and clinical data.

The study protocol was approved by the local Ethics Committee and all participants provided written informed consent.

### 2.2 CSF measurements

CSF samples were collected in 10 mL polypropylene tubes and centrifuged within 2 h (2000 x g, 10 min, room temperature). Immediately after centrifugation, 1 mL aliquots of CSF from each sample were placed in 2 mL polypropylene tubes. All aliquots were stored at −80 °C until analyses. Aβ40, Aβ42, p-tau181 and total tau were analyzed in the Lumipulse G600 II platform from Fujirebio. The cutoff value of Aβ42/Aβ40 that defined CSF-Aβ positivity was ≤ 0.068 (Leitão et al., 2019). For Aβ40, the measurement range was 5–30,000 pg/mL and the lower limit of quantitation (LLOQ) was 4.1 pg/mL [coefficient of variation (CV) = 5%]. For Aβ42, the measurement range was 9–2,335 pg/mL and the LLOQ was 8.5 pg/mL (CV = 4%).

### 2.3 Plasma p-tau217 measurements

Plasma samples were obtained by venipuncture using EDTA-K2 tubes, centrifuged within 3 h of collection, aliquoted into polypropylene tubes, and stored at −80 °*C prior* to measurement, frozen samples were thawed at room temperature for at least 30 min, vortexed for 10 s, and centrifuged at 2,000 g for 5 min. Plasma p-tau217 concentrations were quantified using a chemiluminescent enzyme immunoassay (CLEIA) on the fully automated Lumipulse G600II platform (Fujirebio). The measurement range for the assay was 0.047–10 pg/mL and the LLOQ was 0.047 pg/mL (CV = 10%).

### 2.4 Plasma Aβ measurements

Plasma Aβ42 and Aβ40 concentrations were quantified using ABtest-MS (Araclon Biotech, Zaragoza, Spain), an antibody-free targeted LC–MS method. Briefly, analytes were extracted directly from plasma, and no immunoprecipitation procedure was conducted. Intact Aβ40 and Aβ42 species were measured since no enzymatic digestion was performed. Further details about the analytical procedure and instrumental acquisition parameters are described in the literature (Jang et al., 2021; Allué et al., 2023; Chun et al., 2023; Pascual-Lucas et al., 2023; Fandos et al., 2026). The analytical sensitivity of the assay, together with the accuracy and precision of calibration standards and quality controls from the analytical runs performed in the present study, are provided in Supplementary Tables S1–S3.

### 2.5 APOE genotyping

Genomic DNA was extracted from whole blood using the ReliaPrep Blood gDNA MiniPrep System (Promega, Madison, WI, United States), according to the manufacturer’s instructions. Apolipoprotein E (APOE) genotype was determined using TaqMan SNP genotyping assays (Thermo, Waltham, MA, United States) targeting rs429358 (position 112) and rs7412 (position 158). Genotyping was performed on a StepOnePlus Real-Time PCR System (Applied Biosystems, Waltham, MA, United States) with TaqMan Genotyping Master Mix, following the manufacturer’s protocol. Positive and negative controls were included in each analytical run for quality control.

### 2.6 Statistical analyses

Statistical analyses and graphical representations of the data were conducted using GraphPad Prism v5.03 (GraphPad Software, San Diego, CA, United States), SPSS v18 (IBM, Armonk, NY, United States) and R statistical software (v4.4.2).^1^ To compare different groups, the Chi-square test was used for categorical variables and the Mann–Whitney U test for continuous ones. Associations between plasma biomarker concentrations and CSF Aβ42/Aβ40 were assessed using Spearman rank correlation. Multiple logistic regression models incorporating age, APOE ε4 carrier status (carrier vs. non-carrier; hereafter referred to as APOE), plasma biomarkers, and their combinations were fitted to evaluate diagnostic performance. Receiver Operating Characteristic (ROC) curve analysis was performed and differences in area under the ROC curve (AUC) values were compared by DeLong test. Model calibration was assessed by evaluating the agreement between predicted probabilities and observed proportions using a flexible calibration curve based on Locally Estimated Scatterplot Smoothing (LOESS). Multicollinearity between plasma p-tau217 and Aβ42/Aβ40 was assessed using multiple linear regression adjusting for age and APOE, with CSF Aβ42/Aβ40 specified as the dependent variable. Positive predictive values (PPV) and negative predictive values (NPV) were calculated across a predefined grid of predicted probability thresholds, smoothed using LOESS, and the lower and upper cutoffs were selected as those yielding smoothed PPV and NPV values closest to 95%, respectively. Sensitivity and specificity were calculated in the study cohort using the observed classifications at each cutoff. Methods for internal validation of the models, as well as sensitivity analysis across alternative CSF amyloid prevalences, are described in Supplementary methods.

## 3 Results

### 3.1 Participant characteristics

Baseline characteristics of the study participants, stratified by CSF Aβ status (amyloid positivity defined as CSF Aβ42/Aβ40 ≤ 0.068) are summarized in Table 1. The prevalence of CSF Aβ positivity in the cohort was 50%. CSF Aβ(+) individuals were older than their CSF Aβ(−) counterparts (median [first quartile, Q1–third quartile, Q3]: 73.0 [69.0–76.0] vs. 67.0 [61.0–72.0]; *p* < 0.0001) and performed worse on the Mini-Mental State Examination (MMSE, 27 [24–29] vs. 28 [26–30]; *p* < 0.001). Gender distribution was comparable (*p* = 0.302), whereas the proportion of APOE ε4 carriers and the distribution of APOE ε4 allele count differed significantly between groups (*p* < 0.0001). Significant differences were observed in all plasma and CSF biomarkers between CSF Aβ(+) and CSF Aβ(−) individuals, except for plasma and CSF Aβ40.

**Table 1:** Baseline characteristics of the study participants.

Characteristic	CSF Aβ(+) (*n* = 93)	CSF Aβ(−) (*n* = 93)	*p*-value
Age, years	73.0 [69.0–76.0]	67.0 [61.0–72.0]	**<0.0001**
Female, *N* (%)	55 (59.1)	48 (51.6)	0.302
APOE ε4 carriers, *N* (%)	51 (54.8)	17 (18.3)	**<0.0001**
APOE ε4 alleles, *N* (%)			**<0.0001**
0 alleles ε4	42 (45.2)	76 (81.7)	
1 allele ε4	41 (44.1)	17 (18.3)	
2 alleles ε4	10 (10.8)	0 (0)	
Plasma Aβ40, pg/mL	234.2 [205.2–261.6]	215.0 [199.4–250.2]	0.114
Plasma Aβ42, pg/mL	41.9 [36.7–47.1]	46.3 [41.1–51.9]	**<0.0001**
Plasma Aβ42/Aβ40	0.182 [0.171–0.200]	0.212 [0.195–0.223]	**<0.0001**
Plasma p-tau217, pg/mL	0.418 [0.258–0.713]	0.095 [0.077–0.129]	**<0.0001**
Plasma p-tau217/Aβ42	0.010 [0.006–0.019]	0.002 [0.002–0.003]	**<0.0001**
CSF p-tau181, pg/mL	96.0 [73.5–146.9]	38.9 [29.7–50.5]	**<0.0001**
CSF t-tau, pg/mL	640.0 [476.5–871.0]	282.0 [218.0–367.5]	**<0.0001**
CSF Aβ40, pg/mL	13566.0 [10557.5–16588.5]	12434.0 [9426.0–15819.5]	0.140
CSF Aβ42, pg/mL	641.0 [519.0–779.0]	1282.0 [940.5–1701.5]	**<0.0001**
CSF Aβ42/Aβ40	0.049 [0.042–0.058]	0.109 [0.099–0.115]	**<0.0001**
CSF p-tau181/Aβ42	0.150 [0.104–0.245]	0.029 [0.025–0.035]	**<0.0001**
MMSE, score	27 [24–29]	28 [26–30]	**<0.001**

APOE, apolipoprotein E; MMSE, Mini-Mental State Examination; CSF, cerebrospinal fluid; Aβ, amyloid-beta; p-tau181, phosphorylated tau protein at threonine 181; p-tau217, phosphorylated tau protein at threonine 217; t-tau, total tau protein. Data are median and Q1-Q3 values, except for the variables female and APOE ε4 (carriers or number of alleles) which are the number of cases (%). Differences between groups were tested using Mann–Whitney and chi-square tests, as appropriate. *P*-values in bold correspond to statistically significant results.

### 3.2 Association between plasma biomarkers and CSF Aβ42/Aβ40

Plasma Aβ42/Aβ40 and p-tau217 were both associated with CSF Aβ42/Aβ40 (Spearman *r* = 0.524 and −0.775, respectively; *p* < 0.0001; Supplementary Figure S1). Visual inspection suggested a non-linear relationship between plasma p-tau217 and CSF Aβ42/Aβ40. In contrast, plasma Aβ42/Aβ40 showed an approximately linear relationship. For visualization purposes, linear regression fits with 95% confidence intervals (95% CI) are shown.

### 3.3 Diagnostic performance and grey zone evaluation

A series of logistic regression models were fitted to assess CSF Aβ status using a range of predictors, including demographic covariates (age, APOE), individual plasma biomarkers and multivariable combinations integrating biomarkers with demographic information (Supplementary Table S4). Model performance was evaluated in terms of parsimony (Akaike’s Information Criterion, AIC), explanatory power (Nagelkerke’s pseudo-*R*^2^) and classification accuracy (AUC), in order to identify the optimal balance between goodness-of-fit and discriminative performance (Supplementary Table S5). The best-performing models consistently included p-tau217, either alone or in combination with Aβ measurements and/or demographic variables. ROC curves with corresponding AUC values and 95% CI are shown in Figure 1 and Supplementary Figure S2. AUCs ranged from 0.81 (Aβ42/Aβ40) to 0.97 (models combining p-tau217 with Aβ measurements).

**Figure 1:**
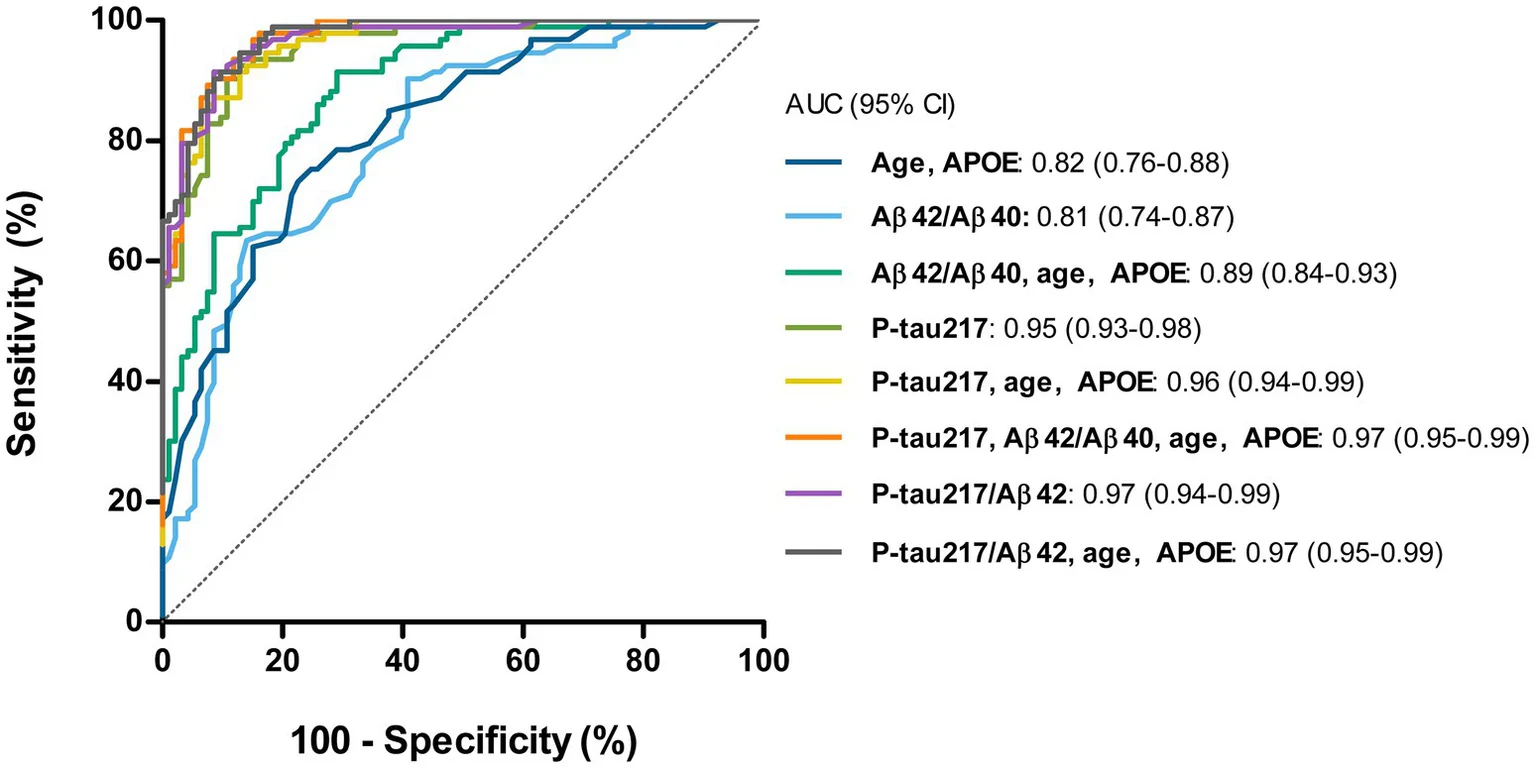
ROC curves corresponding to the logistic regression predictive models assessed.

Model calibration was assessed for the five top-performing models by evaluating the agreement between predicted probabilities and observed proportions of CSF amyloid positivity. The calibration intercept and slope, together with the flexible calibration curves, indicated good overall calibration across the models (Supplementary Figure S3). Diagnostic performance for these five top-performing models was next evaluated with a single cutoff (maximum Youden index). As shown in Supplementary Table S6, only p-tau217/Aβ42 met the 90% sensitivity and specificity required for a confirmatory test. Models incorporating Aβ measurements in addition to p-tau217, yielded significantly higher AUC values than p-tau217 alone, according to DeLong test. Although plasma Aβ42/Aβ40 and p-tau217 are both markers of brain amyloid deposition, no evidence of multicollinearity was observed among predictors (all variance inflation factors <1.2). Predicted probabilities for CSF amyloid positivity of CSF Aβ(+) and Aβ(−) individuals across these models are displayed in Supplementary Figure S4.

Following the initial model evaluation and to maximize classification accuracy, a dual cutoff approach analysis was carried out. Cutoff values, specified PPV and NPV of 95%, were derived by LOESS. Diagnostic performance metrics are shown in Table 2. This approach resulted in grey zones ranging from 12.3 (p-tau217, Aβ42/Aβ40, age, APOE) to 27.4% (p-tau217 alone). Predicted probabilities for CSF Aβ(+) and Aβ(−) individuals across the five top-performing models, highlighting the resulting grey zone in each case, are displayed in Figure 2.

**Table 2:** Diagnostic performance metrics for the five top-performing models calculated with a dual cutoff approach after fixing PPV and NPV at 95%.

Model	Cutoffs	Sensitivity (%)	Specificity (%)	Grey zone (%)
P-tau217	0.20–0.89^a^	96	97	27.4
P-tau217, age, APOE	0.23–0.80	96	96	17.7
P-tau217/Aβ42	0.21–0.75	96	96	13.4
P-tau217, Aβ42/Aβ40, age, APOE	0.34–0.74	96	96	12.3
P-tau217/Aβ42, age, APOE	0.26–0.82	96	97	15.6

APOE, apolipoprotein E; p-tau217, phosphorylated tau protein at threonine 217. ^a^Model probability cutoffs of 0.20 and 0.89 correspond to p-tau217 concentrations of 0.14 and 0.33 pg/mL, respectively.

**Figure 2:**
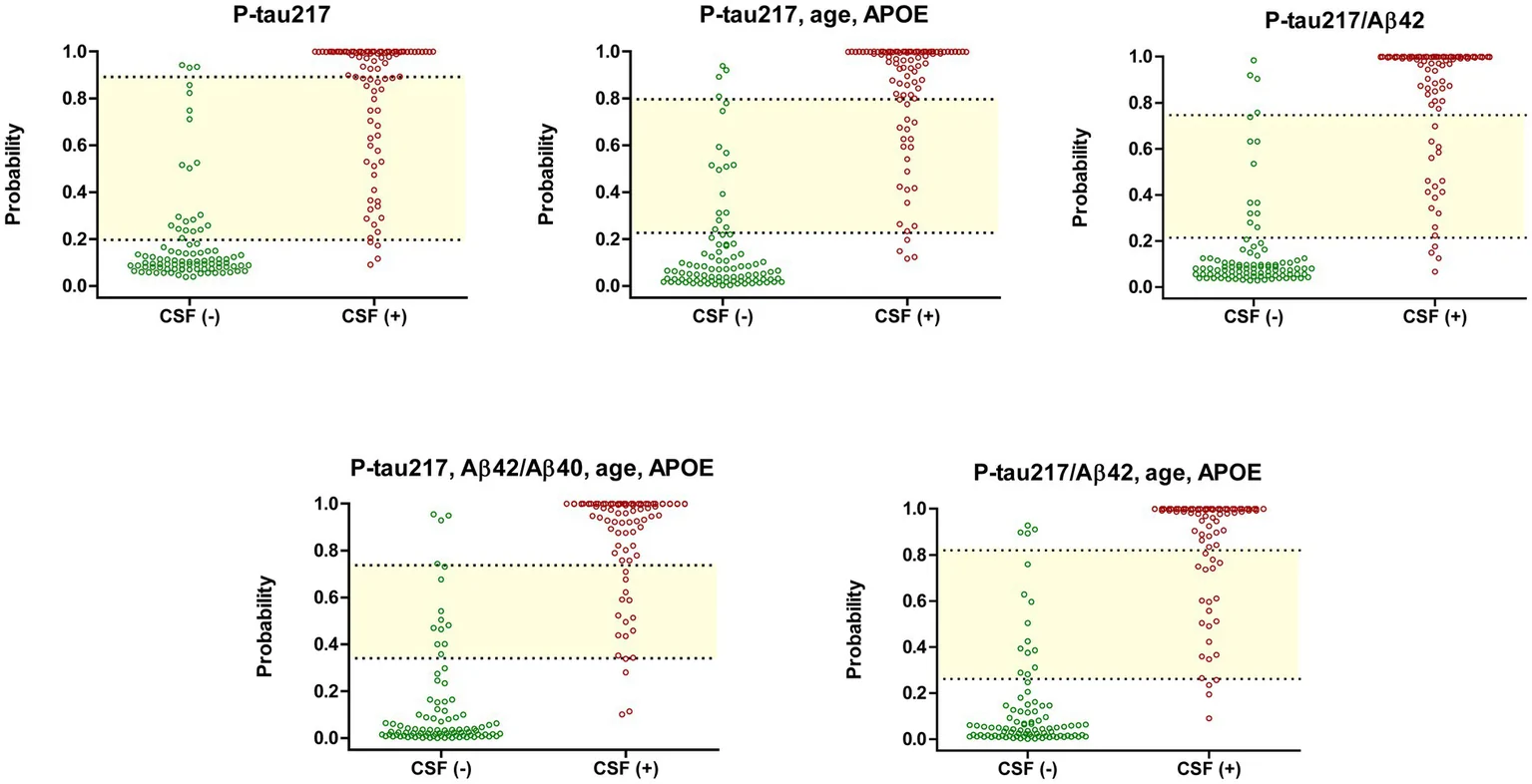
Predicted probability distributions across the five top-performing models, highlighting the area corresponding to the resulting grey zone after setting cutoff points corresponding to PPV and NPV of 95%.

Internal bootstrap validation of these five top-performing models showed that model performance was largely maintained after correction for optimism, with only modest differences between apparent and optimism-corrected estimates (Supplementary Table S7). Overall, these findings indicated limited evidence of overfitting and supported the internal stability of the evaluated models, although the observed bootstrap variability should be considered given the limited sample size.

Sensitivity analyses across alternative CSF amyloid prevalences showed that, at 40 and 60% prevalence, PPV and NPV changed only modestly in the expected direction, while the grey zone proportion remained largely stable (Supplementary Table S8).

### 3.4 Reduction of the p-tau217 grey zone

An additional analysis was performed with the specific aim of balancing the use of a simple first-line diagnostic approach with the need to minimize the indeterminate range and reduce overall costs. Given that p-tau217 has shown the best performance as a single biomarker, together with its broad availability in clinical immunoassays and extensive supporting evidence (Palmqvist et al., 2020; Groot et al., 2022; Ashton et al., 2024; Gonzalez-Ortiz et al., 2024; Ashton et al., 2025; Balogun et al., 2025; Sehrawat et al., 2025; Hibar et al., 2026), subsequent analyses focused on p-tau217 as the initial screening tool.

Applying dual cutoffs for p-tau217 (0.14 pg/mL and 0.33 pg/mL for the lower and upper thresholds, respectively) to maximize predictive values, resulted in an indeterminate grey zone comprising 27.4% of the cohort (*n* = 51). Within this range, 33 individuals (64.7%) were CSF Aβ(+), while 18 (35.3%) were CSF Aβ(−).

In order to resolve indeterminate cases and enable positive or negative classification, an additional set of logistic regression models was evaluated within this sub-population (Supplementary Table S9). The three models with the highest AUC values were selected to evaluate grey zone reduction and included: p-tau217/Aβ42 alone, p-tau217/Aβ42 combined with age and APOE, and p-tau217 combined with Aβ42/Aβ40, age, and APOE. AUC values ranged from 0.86 to 0.89 (Table 3; Figure 3A). Supplementary Table S10 summarizes ROC analysis results for additional evaluated models with lower performance. The dual-cutoff approach was applied again on selected models, and revised grey zones were derived by fixing predictive values at 95%. As shown in Table 3, p-tau217 original indeterminate zone (27.4%) was further reduced 31.4–51.1% of its original size, resulting in final grey zones ranging from 13.4 to 18.8% with this sequential two-step approach. Two models achieved 100% sensitivity and specificity at the expense of somewhat higher percentage of grey zone (16.7–18.8%). Probability distributions for CSF Aβ(+) and Aβ(−) individuals as well as ROC curves for the three selected models are shown in Figure 3B.

**Table 3:** Diagnostic performance metrics in p-tau217 grey zone for the top-performing models calculated with a dual cutoff approach after fixing PPV and NPV at 95%.

Model	AUC (95% CI)	Cutoffs	Sensitivity (%)	Specificity (%)	Grey zone (%)	Reduction (%)^a^
P-tau217/Aβ42	0.86 (0.76–0.96)	0.26–0.85	100	88	13.4	51.1
P-tau217/Aβ42, age, APOE	0.89 (0.80–0.99)	0.23–0.94	100	100	16.7	39.2
P-tau217, Aβ42/Aβ40, age, APOE	0.88 (0.76–1.00)	0.38–0.98	100	100	18.8	31.4

APOE, apolipoprotein E; p-tau217, phosphorylated tau protein at threonine 217. ^a^ Reduction (%) refers to the relative decrease in the proportion of the grey zone compared with that observed for p-tau217 alone (27.4%).

**Figure 3:**
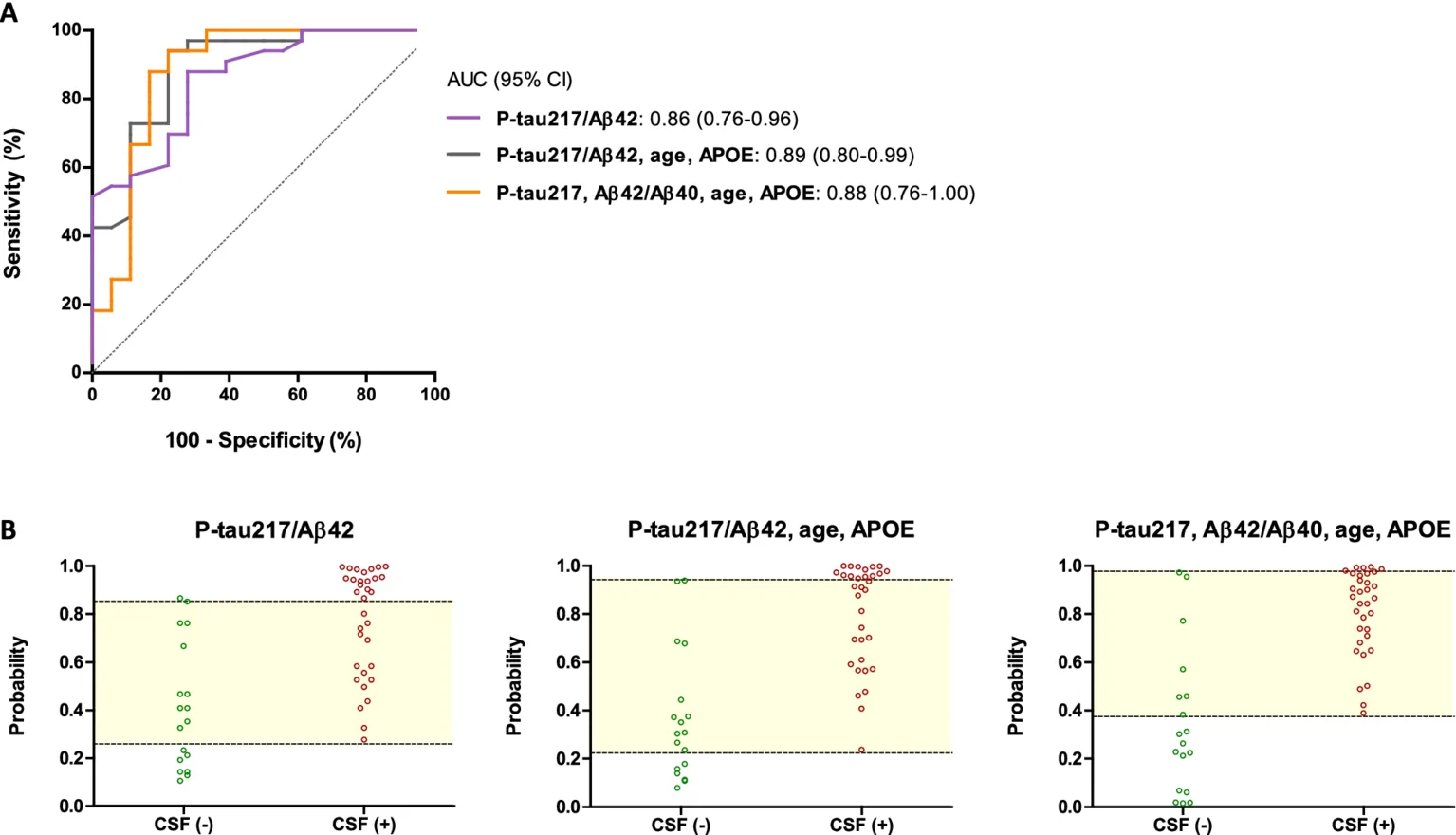
ROC curves **(A)** and predicted probability distributions **(B)** across the top-performing models applied in the analysis of p-tau217 grey zone.

To illustrate the potential impact of increasingly refined biomarker strategies on diagnostic workflows, three complementary scenarios were evaluated (Figure 4). First, when plasma p-tau217 was used as a standalone marker, application of the dual-cutoff approach resulted in an indeterminate range comprising 27.4% of individuals (Table 2), indicating that more than one quarter of the cohort would still require confirmatory CSF or PET assessment. Second, applying multimarker strategies across the full cohort, either using the p-tau217/Aβ42 ratio or combining p-tau217 with Aβ42/Aβ40, age, and APOE, substantially reduced the indeterminate fraction to 12.3–13.4%, corresponding to a potential 51.1–55.1% reduction in the need for CSF or PET tests, at the expense of requiring an additional plasma biomarker measurement in all individuals. Finally, a sequential two-step strategy was explored, in which p-tau217 was first applied to the entire cohort, followed by targeted testing with p-tau217/Aβ42 within the initial indeterminate subgroup. This approach achieved a comparable indeterminate proportion of 13.4% (Table 3), translating into a projected 51.1% reduction in confirmatory procedures. Notably, although predictive values were again fixed at 95%, specificity in this third scenario decreased to 88%, reflecting the misclassification of a single individual as a false positive. In this third scenario, among individuals falling within the p-tau217 grey zone, sequential two-step testing using the p-tau217/Aβ42 ratio correctly reclassified 49% of indeterminate cases, while introducing misclassification in only 2%. The individual classified as false positive by the p-tau217/Aβ42 ratio had CSF Aβ42/Aβ40 value of 0.074, close to the predefined positivity threshold of 0.068.

**Figure 4:**
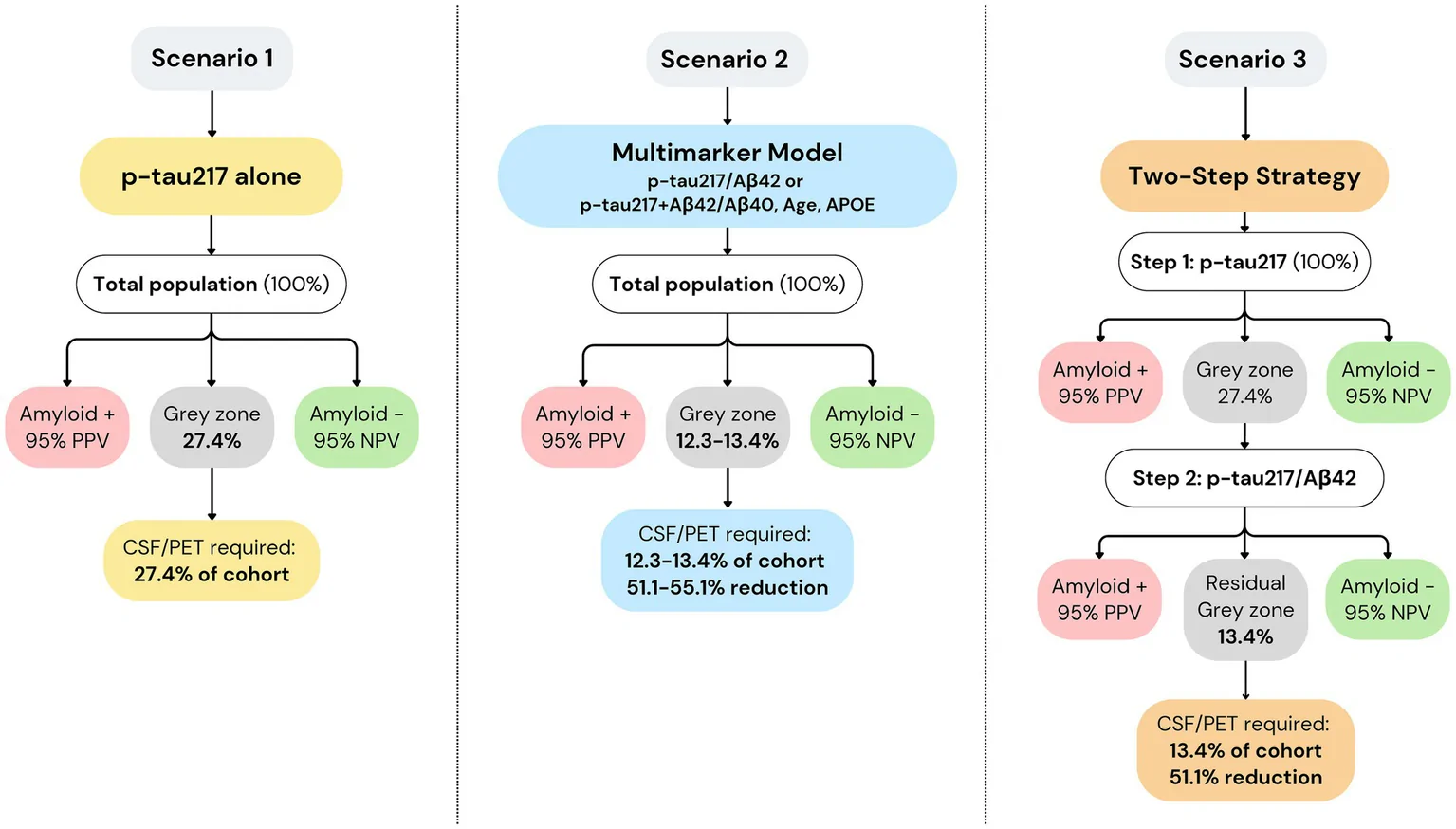
Schematic workflow diagram summarizing the three diagnostic strategies evaluated to optimize amyloid classification using plasma biomarkers. The diagram illustrates (Scenario 1) p-tau217 as a standalone first-line test, (Scenario 2) multimarker integration applied to the entire cohort, and (Scenario 3) a sequential two-step approach in which additional biomarker testing is restricted to individuals within the initial indeterminate range. The workflow highlights how each strategy modifies the size of the grey zone and the downstream need for confirmatory CSF or PET assessment. Detailed methodological specifications are provided in the main text.

This sequential workflow was internally validated using bootstrap resampling, showing that performance was largely maintained after optimism correction. Bias-corrected PPV and NPV were both 95% for the first step and 92 and 96%, respectively, for the second stage (Supplementary Table S11). The final grey zone represented 12.9% of the cohort, corresponding to a 51.5% reduction from the original grey zone, with a bias-corrected misclassification rate of 6% among classified subjects. As expected, given the limited sample size, the bootstrap estimates showed substantial variability, particularly in the second step due to the further reduced sample size.

## 4 Discussion

We evaluated plasma biomarker-based strategies for the detection of cerebral amyloid pathology as defined by CSF Aβ42/Aβ40 in a real-world MCI population. Our primary objective was to reduce the indeterminate zone inherent to dual-cutoff strategies of plasma p-tau217 analysis while maintaining high discriminative performance. At the same time, we aimed to preserve a simple diagnostic workflow based on single-biomarker approaches, given their advantages in terms of cost, assay availability, and ease of implementation, while minimizing the need for confirmatory PET or CSF testing.

Multiple regression models combining amyloid markers with demographic and genetic variables were fitted to assess their ability to identify CSF amyloid status. In these analyses, p-tau217 emerged as the best-performing individual biomarker, in line with previous reports highlighting its strong association with cerebral amyloid pathology (Ashton et al., 2024; Schindler et al., 2024b; Rudolph et al., 2025). The inclusion of additional biomarkers, either by incorporating Aβ42 into a ratio with p-tau217 or by entering plasma Aβ42/Aβ40 as a covariate, resulted in a statistically significant increase in AUC compared with p-tau217 alone. The expanded models also demonstrated better overall fit, reflected by lower AIC values and higher Nagelkerke *R*^2^ indices. While the AUC represents the probability that a randomly selected case will have a higher predicted risk than a randomly selected non-case, AIC and pseudo-*R*^2^ provide complementary information regarding likelihood-based fit and explanatory performance. Their concurrent improvement supports the incremental predictive value of Aβ42 or Aβ42/Aβ40 beyond p-tau217 alone (Niimi et al., 2024; Kang et al., 2025).

Aβ42 has long been recognized as an analytically challenging peptide (Hansson et al., 2021) owing to the hydrophobic nature of its C-terminal sequence, its intrinsic tendency to self-aggregate, and its propensity to adsorb to laboratory surfaces. In plasma, these challenges are further exacerbated by the markedly lower concentrations of Aβ42 and the presence of highly abundant proteins that may interfere with accurate quantification, thereby placing substantial demands on analytical specificity and robustness (Pannee et al., 2021; Verberk et al., 2022). Recent evidence has further highlighted the limitations of Aβ42 quantification in immunoassay-based platforms. Specifically, concerns have been raised regarding the use of Aβ42 in the FDA-cleared Lumipulse assay, where false-positive rates of up to 40% were reported when using the p-tau217/Aβ42 ratio, while this issue was not observed with p-tau217 alone (Algeciras-Schimnich et al., 2026). Together, these findings highlight the challenges associated with Aβ42 quantification in plasma and reinforce the need for robust and reliable methods. MS-based methods provide a valuable alternative for plasma Aβ quantification, offering high analytical specificity and reduced susceptibility to matrix effects. In particular, antibody-free LC–MS approaches, such as ABtest-MS, avoid immunoprecipitation and enzymatic digestion, thereby minimizing potential sources of variability related to cross-reactivity or digestion efficiency.

This analytical robustness has been demonstrated in recent clinical validation study conducted in two independent real-world cohorts of patients with MCI, where MS-derived Aβ42/Aβ40, combined with age and APOE, showed high precision and reproducibility (Allué et al., 2025). In the present study, this MS-based assay was used both to quantify the plasma Aβ42/Aβ40 ratio and to derive Aβ42 concentrations included in the p-tau217/Aβ42 ratio, enabling a consistent and analytically robust integration of Aβ measurements across all evaluated diagnostic models.

Recently, the Global CEO Initiative on AD (CEOi) has recommended that confirmatory tests should achieve ≥90% sensitivity and specificity (Schindler et al., 2024a; Palmqvist et al., 2025b). At a disease prevalence of 50%, such performance corresponds to theoretical PPV and NPV of 90%. When applying a single-cutoff strategy based on the Youden index, all models showed strong diagnostic performance, although only the p-tau217/Aβ42 model achieved both sensitivity and specificity ≥90%. While this highlights the high discriminative capacity of these approaches, especially those combining p-tau217 with Aβ measurements, reliance on a single threshold may be less suitable in clinical practice. In contrast, dual-cutoff approaches offer practical advantages in diagnostic settings by reducing uncertainty around the decision threshold and minimizing misclassification. In line with this framework, the CEOi further recommends that the indeterminate zone should not exceed 20% of individuals (Schindler et al., 2024a), as unresolved results trigger additional visits, testing, and delays in decision-making. In the present work, we implemented more stringent criteria, targeting 95% predictive values; at a 50% prevalence, this corresponds to theoretical sensitivity and specificity of 95%. Under these requirements, the proportion of grey zone results remained below 20% across all evaluated strategies, except for p-tau217 used as a standalone marker.

An important consideration for the implementation of the proposed dual-cutoff strategies is the dependence of PPV and NPV on disease prevalence. Across alternative CSF amyloid prevalences of 40 and 60%, PPV and NPV showed only limited changes in the expected direction, while the proportion of individuals within the grey zone remained largely stable. Nevertheless, the proposed thresholds should be considered proof-of-concept cutoffs derived in the present cohort rather than universally transferable clinical thresholds, and external validation in independent cohorts with varying amyloid prevalence will be necessary to assess their generalizability and clinical applicability.

From a clinical perspective, a key objective is to preserve a simple and scalable first-line diagnostic approach, as single-biomarker strategies offer clear advantages in terms of cost, assay availability, and ease of implementation compared with multimarker testing. In this context, p-tau217 represents a particularly suitable candidate for initial screening, given its strong diagnostic performance, widespread availability in automated immunoassays, and extensive validation across independent cohorts. Accordingly, we focused on the subset of individuals falling within the p-tau217 indeterminate range (27.4%), with the aim of resolving classification by assigning a positive or negative result. Consistent with the findings observed in the full cohort, models incorporating either plasma Aβ42/Aβ40 or Aβ42 with p-tau217, demonstrated superior discriminative ability. The improved discrimination observed after incorporating Aβ peptides is consistent with the complementary biological information provided by these biomarkers (Ashton et al., 2022). Although plasma p-tau217 demonstrates strong performance for identifying amyloid pathology, particularly in symptomatic individuals (Mattsson-Carlgren et al., 2024; Balogun et al., 2025; Schindler et al., 2026), it may not fully capture all variability associated with amyloid status.

This sequential two-step strategy demonstrated that a substantial proportion of indeterminate cases could be potentially reclassified with high certainty. Notably, the discordant case was characterized by CSF Aβ42/Aβ40 values close to the predefined cutoff, highlighting how small variations around the decision threshold can lead to different categorical classifications.

From a practical perspective, our findings support three potential implementation scenarios. First, plasma p-tau217 as a standalone marker within a dual-cutoff framework, yielding three possible outcomes: positive, negative, or indeterminate results. This approach offers the advantage of simplicity, as it relies on the assessment of a single plasma biomarker. However, it would require a high number of additional PET or CSF testing to resolve indeterminate cases, potentially translating into higher costs, delayed clinical decisions, and increased workload for neurology and imaging units. Second, a multimarker strategy incorporating either p-tau217/Aβ42 or p-tau217 combined with Aβ42/Aβ40, age, and APOE is applied to the entire cohort, reducing the grey zone to 12.3–13.4% in a single-step. Although this approach would require additional plasma measurements in all individuals, it could reduce the need for confirmatory PET or CSF testing, with the potential to improve overall diagnostic efficiency. Third, a sequential testing strategy, in which p-tau217 is used as an initial test and second-line assessment is restricted to individuals within the initial indeterminate range. This two-step approach achieves a reduction in the grey zone comparable to the dual-marker strategy applied to the full cohort, while limiting the use of additional plasma biomarkers to a subset of patients. Importantly, the actual impact of these strategies on confirmatory testing, costs, clinical workload, and time to diagnosis was not assessed in the present study and would require prospective evaluation in future studies.

Attempts to reduce the indeterminate range associated with plasma p-tau217 have been previously reported. Recent studies reported a reduction in the proportion of indeterminate results when using the p-tau217/Aβ42 ratio compared with p-tau217 alone (Doecke et al., 2025; Lehmann et al., 2025; Benina et al., 2026). The results observed in our study are consistent with and further support these findings. Importantly, our data additionally show that a comparable reduction in the grey zone can also be achieved by targeting Aβ42 testing to p-tau217-indeterminate individuals only. Sequential approaches have previously been explored, but using p-tau181 rather than Aβ42 (Giacomucci et al., 2026), or through the use of multianalyte panels (Wilson et al., 2025b). By contrasting single-marker, multimarker, and sequential two-step testing approaches within a clinically representative MCI cohort, we clarify the diagnostic and operational trade-offs between maximizing certainty and limiting additional testing to a targeted subset.

This study has several strengths. First, the analyses were conducted in a real-world cohort of patients with MCI evaluated in a secondary-care setting, providing a clinically representative context for assessing the practical implementation of plasma biomarkers. Second, the biomarkers were quantified using analytically robust and fully validated technologies, supporting the reliability of the measurements. Nevertheless, several limitations should be acknowledged. Data on the p-tau217/Aβ42 ratio using Aβ42 quantified by the FDA-cleared Lumipulse assay are not available. We instead used a robust LC–MS-based method for Aβ42 quantification, which provides analytically reliable and consistent estimates that support the validity of the derived ratio, although a direct head-to-head comparison is lacking. Second, the sample size was relatively modest, and the findings have not yet been validated in an independent external cohort. Third, the impact of comorbidities and other clinical factors that may influence plasma biomarker concentrations and diagnostic performance, such as hypertension, diabetes, and renal dysfunction, was not specifically evaluated. Previous studies suggest that plasma biomarker ratios, including Aβ42/Aβ40 and p-tau217/Aβ42, may be relatively robust to certain potential confounding factors, whereas individual biomarkers such as p-tau217 may be more susceptible to these factors (Lehmann et al., 2023; Pichet Binette et al., 2023; Lehmann et al., 2025). Nevertheless, the potential influence of comorbidities and other clinical factors on biomarker concentrations and, consequently, on the diagnostic performance of individual biomarkers and the proposed diagnostic workflows cannot be excluded. Future studies should therefore assess the impact of these factors on plasma biomarker-based diagnostic strategies. Finally, the study population consisted exclusively of Caucasian individuals, and further studies in more diverse populations will be necessary to ensure the generalizability of these findings.

In conclusion, integrated plasma biomarker strategies can meaningfully reduce diagnostic uncertainty in memory-clinic patients with MCI. While plasma p-tau217 remains a highly accurate standalone marker, combining complementary amyloid measures, either directly or through a sequential testing approach, substantially improves classification certainty and limits the proportion of indeterminate results.

## Data Availability

The dataset analyzed during the current study is not publicly available due to participant privacy, ethical restrictions and informed consent limitations, but de-identified data may be available from the corresponding authors upon reasonable request and approval.

## Footnotes

1
https://www.R-project.org
