# Supplementary material for: Plasma biomarker strategies to reduce diagnostic uncertainty in p-tau217–based assessment of CSF amyloid positivity in secondary-care patients with MCI
Source: Front Aging Neurosci. 2026 Sep 28;18:1918304. doi: 10.3389/fnagi.2026.1918304 (PMC13655080; doi:10.3389/fnagi.2026.1918304)
Supplement: Supplementary file 1 [file Data_Sheet_1.docx]

**Supplementary methods**

**Participants in the study**

Definition of cognitive impairment

Cognitive impairment was defined as performance at least one standard deviation below age- and education-adjusted normative data on any test within a comprehensive neuropsychological protocol. This protocol included: the Mini-Mental State Examination (MMSE); Addenbrooke's Cognitive Examination III; digit span (forward and backward); Corsi's block-tapping test (forward and backward); Trail Making Test (parts A and B); Symbol Digit Modalities Test; Stroop Color-Word Interference Test (word reading, color naming, and interference); Tower of London-Drexel version; Boston Naming Test; Free and Cued Selective Reminding Test; Rey-Osterrieth Complex Figure (copy, and 3- and 30-minute recall, and recognition); Judgment of Line Orientation; and the Visual Object and Space Perception Battery (subtests: object decision, progressive silhouettes, position discrimination, and number location).

**Statistical analyses**

Internal validation of the five top-performing models in the full cohort

Internal validation of the logistic regression models was performed using 1,000 bootstrap resamples of the original cohort (N=186). In each bootstrap resample, each model was refitted using the same predictors as in the original analysis and its performance was evaluated both in the bootstrap sample and, without further refitting or recalibration, in the original cohort. Bootstrap optimism was calculated for each performance measure as the difference between performance in the bootstrap sample and performance obtained when the corresponding bootstrap-fitted model was applied to the original cohort. Mean bootstrap optimism was then subtracted from the apparent performance of the original model to obtain optimism-corrected point estimates. Bootstrap distributions of the performance measures were summarized using the median and 2.5^th^–97.5^th^ percentiles. This procedure was applied to discrimination and classification performance measures, including AUC, sensitivity, specificity, PPV, NPV, and the proportion of subjects within the grey zone.

Internal validation of the sequential classification algorithm

The complete sequential classification algorithm was internally validated using 1,000 bootstrap resamples of the original cohort (N=186), sampled with replacement. In each bootstrap resample, the entire algorithm was re-developed. First, a logistic regression model based on p-tau217 was fitted, and low and high probability cutoffs were derived by selecting the pair providing NPV and PPV, respectively, closest to the prespecified 95% targets, with minimization of the grey zone used as a secondary criterion. Subjects with predicted probabilities between these cutoffs constituted the first-stage grey zone. A second logistic regression model based on the p-tau217/Aβ42 ratio was then fitted exclusively within this bootstrap-specific grey zone, and second-stage low and high probability cutoffs were derived using the same procedure.

The complete bootstrap-derived algorithm, including both fitted models and all four probability cutoffs, was subsequently applied without refitting or recalibration to the original cohort. For each performance measure, bootstrap bias was estimated as the mean difference between its value in the bootstrap resample and that obtained when the corresponding bootstrap-derived algorithm was applied to the original cohort. Bias-corrected point estimates were calculated by subtracting this mean bias from the apparent estimate. Bootstrap distributions are summarized as median and 2.5th–97.5th percentiles. Iterations were excluded only when the sequential algorithm could not be formally estimated or its probability cutoffs could not be derived.

Sensitivity analysis across different CSF amyloid positivity prevalences

To assess the dependence of predictive values and grey zone classification on disease prevalence, sensitivity analyses were performed at approximately 40% and 60% CSF amyloid positivity prevalence using prevalence-weighted stratified resampling of the original cohort. For each prevalence scenario, 1,000 samples of N=186 were generated by sampling with replacement separately from the observed CSF Aβ positive and negative subjects, while fixing the number of subjects from each group to reproduce the target prevalence. Specifically, samples contained 74 CSF Aβ positive and 112 CSF Aβ negative subjects for the 40% scenario (39.8% prevalence) and 112 CSF Aβ positive and 74 CSF Aβ negative subjects for the 60% scenario (60.2% prevalence).

The fitted models and the bootstrap-derived dual probability cutoffs obtained from the original cohort (50.0% prevalence) were kept fixed throughout the sensitivity analysis and applied in each resampled dataset. PPV, NPV, and the proportion of subjects within the grey zone were calculated for each resample, with subjects within the grey zone excluded from the calculation of PPV and NPV. Results for the 40% and 60% prevalence scenarios were summarized as the median and 2.5^th^–97.5^th^ percentiles across the 1,000 resamples.

**Figure S1.** Associations between plasma Aβ42/Aβ40 (left) or p-tau217 (right) and CSF Aβ42/Aβ40. A linear fit with 95% CI is depicted for illustrative purposes.

**Figure S2.** Individual ROC curves corresponding to the logistic regression predictive models assessed in the full cohort.

**Figure S3.** Calibration of the five top-performing models in the full cohort. Calibration plots show the relationship between predicted probabilities and observed proportions of CSF amyloid positivity. The calibration intercept and slope are indicated for each model. The diagonal line represents perfect calibration, and the flexible calibration curves were fitted using LOESS.

**Figure S4.** Predicted probability distributions across the five top-performing models in the full cohort. The dashed line indicates the optimal individual cutoff in each case.

**Table S1.** Analytical sensitivity of ABtest-MS.

|  | **ABtest-MS**  **^15^N-Aβ40** | **ABtest-MS**  **^15^N-Aβ42** |
| --- | --- | --- |
| **Sensitivity** |  |  |
| *LLOQ* | 50 pg/mL | 20 pg/mL |
| *Intra-assay RE* | 1.9 to 4.9% | 0.0 to 7.6% |
| *Inter-assay RE* | 4.9% | 3.4% |
| *Intra-assay CV* | 0.5 to 8.8% | 5.2 to 11.9% |
| *Inter-assay CV* | 4.1% | 7.6% |

Abbreviations: CV: coefficient of variation; LLOQ: lower limit of quantification; RE: relative error.

**Table S2.** Accuracy and precision of ABtest-MS for ^15^N-Aβ40 and ^15^N-Aβ42 (Calibration curves analyzed in the present study).

|  | **Concentration of ^15^N-Aβ40 (pg/mL)** | | | | | | |  |
| --- | --- | --- | --- | --- | --- | --- | --- | --- |
|  | **50** | **75** | **100** | **200** | **300** | **400** | **500** | **r** |
| **Run 001** | 51.6 | 75.0 | 94.9 | 195.8 | 288.0 | 437.6 | 506.3 | 0.99801 |
|  | 51.1 | 71.1 | 99.7 | 207.2 | 304.9 | 383.3 | 497.6 |  |
| **Run 002** | 51.9 | 77.9 | 99.2 | 212.9 | 305.2 | 393.4 | 478.6 | 0.99823 |
|  | 49.6 | 70.3 | 94.0 | 202.6 | 304.2 | 414.9 | 488.1 |  |
| **Run 003** | 53.2 | 71.7 | 96.9 | 199.1 | 301.6 | 404.0 | 512.4 | 0.99820 |
|  | 49.1 | 71.2 | 104.1 | 211.4 | 300.7 | 401.4 | 470.4 |  |
| **Run 004** | 53.6 | 75.4 | 100.9 | 204.4 | 288.6 | 400.7 | 517.2 | 0.99849 |
|  | 47.0 | 71.2 | 103.1 | 197.3 | 295.8 | 400.3 | 499.3 |  |
| **Run 005** | 51.8 | 74.0 | 102.5 | 192.8 | 297.8 | 408.9 | 503.6 | 0.99941 |
|  | 48.9 | 75.4 | 97.5 | 195.6 | 300.6 | 400.1 | 513.4 |  |
| **Mean** | 50.8 | 73.3 | 99.3 | 201.9 | 298.7 | 404.5 | 498.7 | 0.99847 |
| **SD** | 2.1 | 2.5 | 3.4 | 6.9 | 6.3 | 14.4 | 15.4 |  |
| **%CV** | 4.1 | 3.5 | 3.5 | 3.4 | 2.1 | 3.6 | 3.1 |  |
| **%RE** | 1.6 | −2.2 | −0.7 | 1.0 | −0.4 | 1.1 | −0.3 |  |

|  | **Concentration of ^15^N-Aβ42 (pg/mL)** | | | | | | |  |
| --- | --- | --- | --- | --- | --- | --- | --- | --- |
|  | **20** | **30** | **40** | **80** | **120** | **160** | **200** | **r** |
| **Run 001** | 20.5 | 31.7 | 42.4 | 87.1 | 118.2 | 171.4 | 200.3 | 0.99512 |
|  | 20.2 | 26.3 | 36.9 | 81.3 | 123.1 | 149.6 | 185.9 |  |
| **Run 002** | 23.4 | 30.9 | 36.9 | 80.5 | 123.0 | 165.3 | 207.0 | 0.99450 |
|  | 18.3 | 27.3 | 37.9 | 82.6 | 118.3 | 157.3 | 200.1 |  |
| **Run 003** |  |  | 48.1 | 81.0 | 126.6 | 158.6 | 204.7 | 0.99660 |
|  | 19.1 | 28.5 | 38.8 | 81.5 | 120.7 | 153.0 | 186.8 |  |
| **Run 004** | 21.6 | 34.0 | 44.4 | 82.8 | 115.7 | 167.6 | 190.1 | 0.99263 |
|  | 17.1 | 27.1 | 39.9 | 77.9 | 112.7 | 159.9 | 202.6 |  |
| **Run 005** | 20.9 | 28.7 | 38.3 | 82.8 | 116.6 | 165.6 | 199.6 | 0.99829 |
|  | 20.8 | 27.9 | 39.9 | 78.1 | 124.4 | 163.2 | 199.6 |  |
| **Mean** | 20.2 | 29.1 | 40.4 | 81.6 | 119.9 | 161.1 | 197.7 | 0.99543 |
| **SD** | 1.9 | 2.5 | 3.6 | 2.6 | 4.4 | 6.7 | 7.4 |  |
| **%CV** | 9.2 | 8.7 | 9.0 | 3.2 | 3.6 | 4.2 | 3.8 |  |
| **%RE** | 1.1 | −2.8 | 0.9 | 2.0 | −0.1 | 0.7 | −1.2 |  |

Abbreviations: CV: coefficient of variation; RE: relative error; SD: standard deviation.

Back-calculated concentrations for ^15^N-Aβ40 (upper) and ^15^N-Aβ42 (lower) at seven concentration levels in the calibration curves, along five analytical runs, are shown. Two calibration curves were analyzed per duplicate, at the beginning and at the end of each analytical sequence. Correlation coefficients correspond to each duplicated calibration curve. The inverse of the theoretical concentration (1/x) was chosen as weighting factor.

**Table S3.** Accuracy and precision of ABtest-MS for ^15^N-Aβ40 and ^15^N-Aβ42 (Quality Control samples analyzed in the present study).

|  | **Concentration of ^15^N-Aβ40 (pg/mL)** | | |
| --- | --- | --- | --- |
|  | **150** | **200** | **400** |
| **Run 001** | 158.4 | 197.9 | 362.5 |
|  | 139.7 | 205.5 | 386.7 |
| **Run 002** | 153.6 | 212.4 | 386.7 |
|  | 149.9 | 204.6 | 368.6 |
| **Run 003** | 140.2 | 208.5 | 404.2 |
|  | 143.2 | 208.2 | 367.2 |
| **Run 004** | 160.0 | 188.5 | 379.5 |
|  | 143.4 | 222.0 | 401.0 |
| **Run 005** | 149.0 | 211.1 | 391.7 |
|  | 145.4 | 197.5 | 379.7 |
| **Mean** | 148.3 | 205.6 | 382.8 |
| **SD** | 7.2 | 9.3 | 14.0 |
| **%CV** | 4.9 | 4.5 | 3.7 |
| **%RE** | −1.2 | 2.8 | −4.3 |

|  | **Concentration of ^15^N-Aβ42 (pg/mL)** | | |
| --- | --- | --- | --- |
|  | **60** | **80** | **160** |
| **Run 001** | 63.2 | 73.0 | 142.2 |
|  | 55.7 | 77.6 | 139.5 |
| **Run 002** | 56.6 | 79.5 | 170.3 |
|  | 63.1 | 80.3 | 144.0 |
| **Run 003** | 55.7 | 78.1 | 162.3 |
|  | 59.0 | 77.1 | 139.1 |
| **Run 004** | 68.0 | 84.9 | 157.8 |
|  | 58.1 | 79.2 | 160.9 |
| **Run 005** | 53.4 | 78.9 | 152.4 |
|  | 56.5 | 81.9 | 145.5 |
| **Mean** | 59.0 | 79.1 | 151.4 |
| **SD** | 4.5 | 3.1 | 10.9 |
| **%CV** | 7.6 | 4.0 | 7.2 |
| **%RE** | −1.7 | −1.2 | −5.4 |

Abbreviations: CV: coefficient of variation; RE: relative error; SD: standard deviation.

Back-calculated concentrations for ^15^N-Aβ40 (upper) and ^15^N-Aβ42 (lower) in Quality Control samples, along five analytical runs, are shown. Three concentration levels were assayed: low (3x Lower Limit of Quantitation [LLOQ]), mid and high, for both analytes. Six Quality Control samples, two per concentration level, were included in each analytical run.

**Table S4.** Regression coefficients and predictor definitions for the logistic regression models.

| **Model** | **Predictor** | **B** | **95% CI** | **Definition** |
| --- | --- | --- | --- | --- |
| **Age, APOE** | Intercept | −11.72 | N/A | N/A |
|  | Age | 0.16 | 0.10-0.22 | Years |
|  | APOE | 2.04 | 1.26-2.81 | 0 = non-carrier; 1 = carrier |
| **Aβ42/Aβ40** | Intercept | 11.41 | N/A | N/A |
|  | Aβ42/Aβ40 | −58.06 | −75.98- −40.14 | Plasma Aβ42 concentration / plasma Aβ40 concentration |
| **Aβ42/Aβ40, age, APOE** | Intercept | −0.83 | N/A | N/A |
|  | Aβ42/Aβ40 | −49.69 | −69.09- −30.29 | Plasma Aβ42 concentration / plasma Aβ40 concentration |
|  | Age | 0.14 | 0.08-0.21 | Years |
|  | APOE | 1.69 | 0.83-2.55 | 0 = non-carrier; 1 = carrier |
| **P-tau217** | Intercept | −3.99 | N/A | N/A |
|  | p-tau217 | 18.68 | 13.02-24.33 | Continuous |
| **P-tau217, age, APOE** | Intercept | −12.05 | N/A | N/A |
|  | p-tau217 | 15.34 | 9.49-21.18 | Continuous |
|  | Age | 0.11 | 0.01-0.21 | Years |
|  | APOE | 2.00 | 0.86-3.14 | 0 = non-carrier; 1 = carrier |
| **P-tau217, Aβ42/Aβ40, age, APOE** | Intercept | −0.30 | N/A | N/A |
|  | p-tau217 | 15.88 | 9.38-22.38 | Continuous |
|  | Aβ42/Aβ40 | −49.37 | −78.85- −19.89 | Plasma Aβ42 concentration / plasma Aβ40 concentration |
|  | Age | 0.08 | −0.03-0.19 | Years |
|  | APOE | 1.89 | 0.63-3.14 | 0 = non-carrier; 1 = carrier |
| **P-tau217/Aβ42** | Intercept | −4.63 | N/A | N/A |
|  | P-tau217/Aβ42 | 993.21 | 687.03-1299.39 | Plasma p-tau217 concentration / plasma Aβ42 concentration |
| **P-tau217/Aβ42, age, APOE** | Intercept | −14.09 | N/A | N/A |
|  | P-tau217/Aβ42 | 817.10 | 501.20-1132.99 | Plasma p-tau217 concentration / plasma Aβ42 concentration |
|  | Age | 0.13 | 0.02-0.25 | Years |
|  | APOE | 1.90 | 0.64-3.16 | 0 = non-carrier; 1 = carrier |

Abbreviations; APOE: apolipoprotein E; p-tau217: phosphorylated tau protein at threonine 217; CI: confidence interval.

**Table S5.** Goodness-of-fit and discrimination metrics for the assessed models.

|  | **Goodness-of-fit** | | **Discrimination** |
| --- | --- | --- | --- |
| **Model** | **AIC** | **R^2^** | **AUC (95% CI)** |
| **Age, APOE** | 203.2 | 0.392 | 0.82 (0.76-0.88) |
| **Aβ42/Aβ40** | 203.2 | 0.382 | 0.81 (0.74-0.87) |
| **Aβ42/Aβ40, age, APOE** | 169.7 | 0.553 | 0.89 (0.84-0.93) |
| **P-tau217** | 114.5 | 0.729 | 0.95 (0.93-0.98) |
| **P-tau217, age, APOE** | 102.4 | 0.779 | 0.96 (0.94-0.99) |
| **P-tau217, Aβ42/Aβ40, age, APOE** | 90.8 | 0.819 | 0.97 (0.95-0.99) |
| **P-tau217/Aβ42** | 97.6 | 0.782 | 0.97 (0.94-0.99) |
| **P-tau217/Aβ42, age, APOE** | 88.4 | 0.820 | 0.97 (0.95-0.99) |

Abbreviations; APOE: apolipoprotein E; p-tau217: phosphorylated tau protein at threonine 217; AIC: Akaike’s Information Criterion; R^2^: Nagelkerke´s pseudo-R^2^; AUC: area under the receiver operating characteristic curve; CI: confidence interval.

**Table S6.** Diagnostic performance metrics for the five top-performing models calculated at the maximum Youden’s index.

| **Model** | **Cutoff** | **Sensitivity (%)** | **Specificity (%)** | **PPV (%)** | **NPV (%)** | **Accuracy (%)** | **AUC (95% CI)** | **P value^a^** |
| --- | --- | --- | --- | --- | --- | --- | --- | --- |
| **P-tau217** | 0.285^b^ | 92 | 87 | 88 | 92 | 90 | 0.95 (0.91-0.98) | N/A |
| **P-tau217, age, APOE** | 0.334 | 92 | 86 | 87 | 92 | 87 | 0.96 (0.93-0.99) | 0.239 |
| **P-tau217, Aβ42/Aβ40, age, APOE** | 0.277 | 98 | 84 | 86 | 98 | 91 | 0.97 (0.94-0.99) | 0.047 |
| **P-tau217/Aβ42** | 0.377 | 91 | 91 | 91 | 91 | 91 | 0.97 (0.93-0.99) | 0.007 |
| **P-tau217/Aβ42, age, APOE** | 0.329 | 95 | 87 | 88 | 94 | 91 | 0.97 (0.94-0.99) | 0.019 |

Abbreviations; APOE: apolipoprotein E; p-tau217: phosphorylated tau protein at threonine 217; PPV: positive predictive value; NPV: negative predictive value; AUC: area under the receiver operating characteristic curve. ^a^P values were obtained using the DeLong test, with the p-tau217 model considered as the reference model. No adjustment for multiple comparisons was applied. ^b^Model probability cutoff of 0.285 corresponds to a p-tau217 concentration of 0.17 pg/mL.

**Table S7.** Bootstrap internal validation and optimism-corrected performance of the five top-performing models in the full cohort.

**Model: P-tau217**

| **Parameter** | **Bootstrap estimate** | **2.5^th^ percentile** | **97.5^th^ percentile** | **Optimism corrected** |
| --- | --- | --- | --- | --- |
| AUC | 0.95 | 0.92 | 0.98 | 0.95 |
| Low probability cutoff | 0.19 | 0.13 | 0.29 |  |
| High probability cutoff | 0.85 | 0.47 | 0.92 |  |
| PPV (%) | 95 | 91 | 100 | 95 |
| NPV (%) | 95 | 92 | 98 | 94 |
| Sensitivity (%) | 95 | 90 | 98 | 93 |
| Specificity (%) | 95 | 92 | 100 | 95 |
| Grey zone (%) | 24.2 | 7.5 | 40.4 |  |

**Model: P-tau217/Aβ42**

| **Parameter** | **Bootstrap estimate** | **2.5^th^ percentile** | **97.5^th^ percentile** | **Optimism corrected** |
| --- | --- | --- | --- | --- |
| AUC | 0.97 | 0.94 | 0.98 | 0.97 |
| Low probability cutoff | 0.22 | 0.13 | 0.37 |  |
| High probability cutoff | 0.77 | 0.43 | 0.90 |  |
| PPV (%) | 95 | 93 | 97 | 96 |
| NPV (%) | 95 | 93 | 97 | 95 |
| Sensitivity (%) | 95 | 91 | 97 | 94 |
| Specificity (%) | 95 | 92 | 98 | 96 |
| Grey zone (%) | 15.6 | 3.6 | 30.6 |  |

**Model: P-tau217, age, APOE**

| **Parameter** | **Bootstrap estimate** | **2.5^th^ percentile** | **97.5^th^ percentile** | **Optimism corrected** |
| --- | --- | --- | --- | --- |
| AUC | 0.96 | 0.94 | 0.98 | 0.96 |
| Low probability cutoff | 0.22 | 0.13 | 0.43 |  |
| High probability cutoff | 0.80 | 0.53 | 0.90 |  |
| PPV (%) | 95 | 92 | 98 | 95 |
| NPV (%) | 95 | 92 | 98 | 94 |
| Sensitivity (%) | 95 | 91 | 98 | 93 |
| Specificity (%) | 95 | 92 | 98 | 95 |
| Grey zone (%) | 18.8 | 4.8 | 34.4 |  |

**Model: P-tau217/Aβ42, age, APOE**

| **Parameter** | **Bootstrap estimate** | **2.5^th^ percentile** | **97.5^th^ percentile** | **Optimism corrected** |
| --- | --- | --- | --- | --- |
| AUC | 0.97 | 0.95 | 0.99 | 0.97 |
| Low probability cutoff | 0.30 | 0.18 | 0.46 |  |
| High probability cutoff | 0.79 | 0.46 | 0.89 |  |
| PPV (%) | 95 | 92 | 98 | 94 |
| NPV (%) | 95 | 92 | 97 | 93 |
| Sensitivity (%) | 95 | 91 | 97 | 92 |
| Specificity (%) | 95 | 92 | 99 | 94 |
| Grey zone (%) | 12.9 | 1.6 | 25.3 |  |

**Model: P-tau217, Aβ42/Aβ40, age, APOE**

| **Parameter** | **Bootstrap estimate** | **2.5^th^ percentile** | **97.5^th^ percentile** | **Optimism corrected** |
| --- | --- | --- | --- | --- |
| AUC | 0.97 | 0.95 | 0.99 | 0.97 |
| Low probability cutoff | 0.35 | 0.19 | 0.50 |  |
| High probability cutoff | 0.74 | 0.49 | 0.91 |  |
| PPV (%) | 95 | 92 | 97 | 95 |
| NPV (%) | 95 | 93 | 97 | 94 |
| Sensitivity (%) | 95 | 91 | 97 | 94 |
| Specificity (%) | 95 | 93 | 98 | 95 |
| Grey zone (%) | 12.4 | 1.5 | 28.0 |  |

Values shown in these tables are bootstrap performance estimates, summarized as median and 2.5^th^-97.5^th^ percentiles across 1,000 bootstrap resamples, and optimism-corrected performance estimates. Bootstrap optimism was calculated as the mean difference between performance in each bootstrap sample and performance of the corresponding bootstrap-fitted model when applied to the original cohort. Optimism-corrected estimates were obtained by subtracting the mean bootstrap optimism from the corresponding apparent estimates.

Abbreviations; APOE: apolipoprotein E; p-tau217: phosphorylated tau protein at threonine 217; PPV: positive predictive value; NPV: negative predictive value; AUC: area under the receiver operating characteristic curve.

**Table S8.**  Sensitivity analyses of the five top-performing models in the full cohort at alternative prevalence values.

**Model: P-tau217**

| **Parameter (%)** | **Prevalence (%)** | **Bootstrap estimate** | **2.5^th^ percentile** | **97.5^th^ percentile** |
| --- | --- | --- | --- | --- |
| PPV (%) | 40 | 91 | 84 | 98 |
|  | 60 | 96 | 91 | 100 |
| NPV (%) | 40 | 97 | 93 | 100 |
|  | 60 | 92 | 85 | 98 |
| Grey zone (%) | 40 | 21.5 | 16.1 | 27.4 |
|  | 60 | 23.7 | 17.7 | 29.6 |

**Model: P-tau217/Aβ42**

| **Parameter (%)** | **Prevalence (%)** | **Bootstrap estimate** | **2.5^th^ percentile** | **97.5^th^ percentile** |
| --- | --- | --- | --- | --- |
| PPV (%) | 40 | 95 | 88 | 99 |
|  | 60 | 98 | 94 | 100 |
| NPV (%) | 40 | 97 | 93 | 100 |
|  | 60 | 93 | 86 | 98 |
| Grey zone (%) | 40 | 13.4 | 9.1 | 18.3 |
|  | 60 | 14.5 | 9.1 | 19.4 |

**Model: P-tau217, age, APOE**

| **Parameter (%)** | **Prevalence (%)** | **Bootstrap estimate** | **2.5^th^ percentile** | **97.5^th^ percentile** |
| --- | --- | --- | --- | --- |
| PPV (%) | 40 | 92 | 85 | 98 |
|  | 60 | 96 | 92 | 100 |
| NPV (%) | 40 | 97 | 93 | 100 |
|  | 60 | 93 | 86 | 98 |
| Grey zone (%) | 40 | 17.7 | 12.4 | 23.1 |
|  | 60 | 18.8 | 14.0 | 24.2 |

**Model: P-tau217/Aβ42, age, APOE**

| **Parameter (%)** | **Prevalence (%)** | **Bootstrap estimate** | **2.5^th^ percentile** | **97.5^th^ percentile** |
| --- | --- | --- | --- | --- |
| PPV (%) | 40 | 92 | 85 | 98 |
|  | 60 | 97 | 92 | 100 |
| NPV (%) | 40 | 96 | 92 | 99 |
|  | 60 | 91 | 85 | 97 |
| Grey zone (%) | 40 | 12.9 | 8.6 | 17.2 |
|  | 60 | 14.5 | 9.1 | 19.4 |

**Model: P-tau217, Aβ42/Aβ40, age, APOE**

| **Parameter (%)** | **Prevalence (%)** | **Bootstrap estimate** | **2.5^th^ percentile** | **97.5^th^ percentile** |
| --- | --- | --- | --- | --- |
| PPV (%) | 40 | 94 | 89 | 99 |
|  | 60 | 98 | 94 | 100 |
| NPV (%) | 40 | 97 | 93 | 100 |
|  | 60 | 93 | 87 | 98 |
| Grey zone (%) | 40 | 12.9 | 8.1 | 17.7 |
|  | 60 | 12.9 | 8.6 | 18.8 |

Values shown in these tables are bootstrap performance estimates, summarized as median and 2.5th–97.5th percentiles across 1,000 prevalence-weighted stratified resamples of the original cohort, using fixed bootstrap-derived probability cutoffs. Target Aβ-positivity prevalences of 40% and 60% corresponded to actual prevalences of 39.8% (74/186) and 60.2% (112/186), respectively.

Abbreviations; APOE: apolipoprotein E; p-tau217: phosphorylated tau protein at threonine 217; PPV: positive predictive value; NPV: negative predictive value.

**Table S9.** Regression coefficients and predictor definitions for the second-stage logistic regression models.

| **Model** | **Predictor** | **B** | **95% CI** | **Definition** |
| --- | --- | --- | --- | --- |
| **Aβ42/Aβ40** | Intercept | 13.10 | N/A | N/A |
|  | Aβ42/Aβ40 | −63.56 | −103.22-23.59 | Plasma Aβ42 concentration / plasma Aβ40 concentration |
| **Aβ42/Aβ40, age, APOE** | Intercept | 9.06 | N/A | N/A |
|  | Aβ42/Aβ40 | -59.00 | −101.23-16.78 | Plasma Aβ42 concentration / plasma Aβ40 concentration |
|  | Age | 0.03 | −0.11-0.18 | Years |
|  | APOE | 1.49 | 0.00-2.98 | 0 = non-carrier; 1 = carrier |
| **P-tau217** | Intercept | −2.46 | N/A | N/A |
|  | P-tau217 | 14.11 | 2.99-25.22 | Continuous |
| **P-tau217, age, APOE** | Intercept | −3.01 | N/A | N/A |
|  | P-tau217 | 12.99 | 1.00-24.98 | Continuous |
|  | Age | 0.00 | −0.14-0.14 | Years |
|  | APOE | 1.57 | 0.13-3.00 | 0 = non-carrier; 1 = carrier |
| **P-tau217, Aβ42/Aβ40, age, APOE** | Intercept | 12.89 | N/A | N/A |
|  | P-tau217 | 17.11 | 1.99-32.24 | Continuous |
|  | Aβ42/Aβ40 | −68.01 | −116.05-−19.98 | Plasma Aβ42 concentration / plasma Aβ40 concentration |
|  | Age | −0.05 | -0.22-0.13 | Years |
|  | APOE | 1.70 | −0.18-3.12 | 0 = non-carrier; 1 = carrier |
| **P-tau217/Aβ42** | Intercept | −4.93 | N/A | N/A |
|  | P-tau217/Aβ42 | 1167.45 | 481.58-1853.32 | Plasma p-tau217 concentration / plasma Aβ42 concentration |
| **P-tau217/Aβ42, age, APOE** | Intercept | −7.70 | N/A | N/A |
|  | P-tau217/Aβ42 | 1131.98 | 409.34-1854.63 | Plasma p-tau217 concentration / plasma Aβ42 concentration |
|  | Age | 0.03 | −0.12-0.19 | Years |
|  | APOE | 1.47 | −0.20-3.15 | 0 = non-carrier; 1 = carrier |

Abbreviations; APOE: apolipoprotein E; p-tau217: phosphorylated tau protein at threonine 217; CI: confidence interval.

**Table S10 -** ROC analyses of additional models assessed in the p-tau217 grey zone.

| **Model** | **AUC (95% CI)** |
| --- | --- |
| **P-tau217** | 0.74 (0.60-0.89) |
| **Aβ42/Aβ40** | 0.76 (0.63-0.89) |
| **P-tau217, age, APOE** | 0.80 (0.67-0.94) |
| **Aβ42/Aβ40, age, APOE** | 0.83 (0.72-0.95) |

Abbreviations; APOE: apolipoprotein E; p-tau217: phosphorylated tau protein at threonine 217; AUC: area under the receiver operating characteristic curve; CI: confidence interval.

**Table S11 -** Internal validation of the sequential classification algorithm.

| **Parameter** | **Bootstrap estimate** | **2.5^th^ percentile** | **97.5^th^ percentile** | **Optimism corrected** |
| --- | --- | --- | --- | --- |
| **Stage 1 — P-tau217** |  |  |  |  |
| Low probability cutoff | 0.19 | 0.09 | 0.30 |  |
| High probability cutoff | 0.86 | 0.49 | 0.95 |  |
| PPV (%) | 95 | 93 | 97 | 95 |
| NPV (%) | 95 | 93 | 96 | 95 |
| Sensitivity (%) | 95 | 90 | 97 | 93 |
| Specificity (%) | 95 | 91 | 98 | 95 |
| Grey zone (%) | 25.3 | 6.5 | 48.4 | 26.5 |
| **Stage 2 — P-tau217/Aβ42** |  |  |  |  |
| Low probability cutoff | 0.31 | 0.12 | 0.71 |  |
| High probability cutoff | 0.81 | 0.51 | 0.93 |  |
| PPV (%) | 95 | 90 | 100 | 92 |
| NPV (%) | 100 | 60 | 100 | 96 |
| Sensitivity (%) | 100 | 60 | 100 | 97 |
| Specificity (%) | 95 | 50 | 100 | 84 |
| Grey zone (%) | 44.1 | 5.9 | 77.2 | 48.5 |
| **Complete sequential algorithm** |  |  |  |  |
| Final grey zone (% of total cohort) | 11.8 | 0.8 | 24.2 | 12.9 |
| Grey zone reduction (%) | 55.9 | 22.9 | 94.2 | 51.5 |

Abbreviations; p-tau217: phosphorylated tau protein at threonine 217; PPV: positive predictive value; NPV: negative predictive value.
